# Standardized Selective Clipping for Persistent Immediate Bleeding after Cold Snare Polypectomy of Small Colorectal Polyps: A Prospective Cohort Study

**DOI:** 10.1055/a-2937-7891

**Published:** 2026-09-30

**Authors:** Dandan Zhao, Xiaoyan Zhang, Feng Gu, Rui Guo, Tianxu Jia, Shuang Yang, Wenwen Shen, Xiaocui Li, Ding Peng, Ziyu Liu, Hui-Hong Zhai

**Affiliations:** 1 Department of Gastroenterology, National Clinical Research Center for Geriatric Diseases 71044 Xuanwu Hospital, Capital Medical UniversityBeijingBeijingChina

**Keywords:** Endoscopy lower GI tract, Lower GI bleeding, Endoscopic resection (polypectomy, ESD, EMRc, …), Polyps/adenomas/…

## Abstract

**Background and Study Aims** Delayed bleeding after cold snare polypectomy of 4-9 mm nonpedunculated colorectal polyps is rare, but persistent immediate bleeding is relatively common and lacks a standardized management framework. We prospectively evaluated 30-day outcomes of a standardized selective clipping protocol for persistent immediate bleeding after cold snare polypectomy.

**Patients and Methods** In this prospective, single-center cohort study, consecutive adults with at least one eligible 4-9 mm nonpedunculated colorectal polyp resected by cold snare polypectomy between January 1 and December 31, 2024 were enrolled. All bleeding defects were irrigated and observed for 60s, and clips were applied only for persistent oozing according to a standardized selective clipping protocol. The primary endpoint was delayed bleeding within 30 days after polypectomy.

**Results** Among 2176 patients with 4343 polyps, 315 patients (14.5%) and 369 lesions (8.5%) required clipping for persistent immediate bleeding. Delayed bleeding occurred in 5 patients (0.23%), all confirmed by repeat colonoscopy. Patient-level delayed bleeding rates were 0.63% (2/315) in the selective-clipping group and 0.16% (3/1861) in the group without clipping. Lesion-level delayed bleeding rates were 0.54% (2/369) in clip-treated lesions and 0.13% (5/3974) in unclipped lesions. Exploratory analysis suggested chronic kidney disease and five or more synchronous polyps as candidate risk factors.

**Conclusions** Under a standardized selective clipping protocol, delayed bleeding after cold snare polypectomy of 4-9 mm nonpedunculated colorectal polyps was infrequent in both groups, and all confirmed bleeding events were successfully managed endoscopically.

## Introduction

Cold snare polypectomy (CSP) is recommended by both the American Gastroenterological Association and the European Society of Gastrointestinal Endoscopy (ESGE) as the preferred resection technique for most colorectal polyps smaller than 10 mm because it avoids electrocautery-associated deep mural injury, while preserving excellent resection efficacy. 1 2 For these small lesions, current guidance does not support routine prophylactic clip closure of post-polypectomy defects smaller than 20 mm, because delayed bleeding is uncommon and any benefit of clipping appears concentrated in larger proximal mucosal defects after endoscopic mucosal resection rather than in small CSP wounds. 1 2

Delayed post-polypectomy bleeding (DPPB) after CSP of small colorectal polyps is uncommon and has been consistently reported at approximately 0.1-0.3% for 4-9 mm lesions in large cohorts. 3 4 5 By comparison, immediate post-polypectomy bleeding (IPPB) is more frequent but less consistently reported, because its definition varies across studies and prospective data remain limited, with lesion-level rates ranging from 1.1% to 13.0% depending on whether IPPB is defined as bleeding lasting >30 s, bleeding persisting beyond 60s, or bleeding requiring endoscopic hemostasis. 6 7 8 9 10 Most immediate oozing after CSP is self-limited and should not be regarded as a surrogate endpoint for DPPB prevention. This heterogeneity limits cross-study comparability and underscores the need for a reproducible threshold to guide management of persistent intraprocedural bleeding.

This scenario is clinically important because the key decision after CSP is often how to manage the subset of defects with persistent IPPB, not whether every small defect should be clipped. Current guidelines do not recommend routine prophylactic clipping for small defects, 11 12 13 yet they provide limited procedural detail on how persistent IPPB should be managed in daily practice. As a result, treatment is frequently based on endoscopist judgment, which may introduce subjectivity and interoperator variability. Prospective data describing standardized management of persistent IPPB after small-polyp CSP remain limited.

We therefore conducted a prospective cohort study to describe the incidence, clinical course, and 30-day outcomes of 4-9 mm colorectal polyps managed using a standardized selective-clip protocol in which bleeding defects were continuously irrigated and observed for 60s, with clips applied only for persistent IPPB. This study was designed to describe clinical outcomes under this standardized management pathway, rather than to evaluate the preventive efficacy of clipping. A secondary objective was to explore patient-level risk factors for DPPB.

## Patients and Methods

### Study Design and Ethics

This prospective, single-center cohort study was conducted at Xuanwu Hospital, Capital Medical University (Beijing, China), between January 1 and December 31, 2024. Consecutive adults undergoing colonoscopy were screened for eligibility during the study period. The protocol was approved by the Ethics Committee of Xuanwu Hospital, Capital Medical University (approval No. 20220913), all participants provided written informed consent, and the study was registered at the Chinese Clinical Trial Registry (ChiCTR2400087007). The study was conducted in accordance with the Declaration of Helsinki.

### Participants

Adults aged 18 years or older were eligible if they underwent CSP of at least one 4-9 mm nonpedunculated colorectal polyp during routine colonoscopic practice. Exclusion criteria were lesions suspicious for malignancy, inflammatory bowel disease, familial adenomatous polyposis or other polyposis syndromes, and inability to provide informed consent. When multiple eligible lesions were removed during the same examination, all were recorded.

### Endoscopic Procedure and Hemostasis Algorithm

At our center, CSP is the standard resection method for nonpedunculated 4-9 mm lesions without endoscopic features suspicious for deep invasion. Colonoscopies were performed with high-definition video colonoscopes (Olympus, Fujifilm, and Aohua platforms). Dedicated cold snares with 10-mm or 15-mm loop diameters (Micro-Tech, Anrei, or equivalent) were used. Lesions were resected with CSP using a 1-3 mm margin of normal mucosa whenever feasible. If visible residual tissue remained after the first cut, additional cold snare resection was performed until complete endoscopic removal was achieved. The mucosal defect was then carefully inspected.

If bleeding was present at the resection site, timing began after the mucosal defect was clearly visualized. The defect was kept under direct endoscopic view and continuously irrigated with sterile water for 60s; suction was used as needed to clear pooled blood and maintain visualization. This timed assessment was applied only to defects with visible bleeding after CSP; defects without bleeding were not subjected to mandatory 60-second observation. Persistent visible oozing beyond 60s was the protocol-defined indication for clip placement, and clips were deployed by the performing endoscopist until adequate hemostasis was achieved. Defects without bleeding or with bleeding that ceased within 60s were left unclipped. This 60-second irrigation-and-observation rule constituted the standardized post-CSP bleeding management protocol at our center.

### Definitions and Grouping

IPPB was defined as bleeding observed from the mucosal defect immediately after CSP. Persistent IPPB was defined as oozing that continued after 60s of continuous irrigation/observation and therefore required clip placement under the standardized protocol. DPPB was defined as colorectal bleeding within 30 days after CSP that resulted in emergency presentation, hospitalization, or repeat endoscopic confirmation of bleeding or stigmata of recent hemorrhage at a CSP site. Severe DPPB was defined as DPPB associated with a hemoglobin drop of >20 g/L, transfusion, repeat therapeutic endoscopy, angiographic intervention, or surgery. The primary analysis was prespecified at the patient level: patients were assigned to the selective-clip group if at least one lesion developed persistent IPPB and underwent clip placement; all remaining patients were assigned to the no-clip group. Secondary lesion-level analyses classified each resection site as clip-treated or unclipped according to whether clips were applied for persistent IPPB. In patients with postprocedural bleeding and multiple resection sites, culprit lesions were adjudicated at repeat endoscopy according to predefined stigmata of recent hemorrhage: active bleeding, an exposed vessel, or an adherent clot persisting after targeted irrigation. If more than one defect met these criteria, each was recorded as a culprit lesion. When no single definitive culprit lesion could be identified, adjudication incorporated the intraprocedural IPPB location together with lesion size, morphology, and anatomic location. All DPPB cases were reviewed independently by two endoscopists, with disagreements resolved by consensus involving a third reviewer.

### Outcomes and Follow-up

The primary endpoint was DPPB. Secondary outcomes were prespecified as bleeding-related outcomes (hematochezia or melena without confirmed DPPB, time to DPPB onset, bleeding-related length of stay, and lesion-level DPPB) and other safety outcomes (perforation and post-polypectomy fever). Follow-up was conducted by telephone on days 1, 3, and 7, and all emergency visits, rehospitalizations, and endoscopic findings within 30 days were ascertained from the electronic medical record.

### Data Collection and Statistical Analysis

Baseline demographics, comorbidities, antithrombotic therapy, bowel preparation quality, polyp characteristics (location, Paris morphology, size estimated by comparison with the open snare), polypectomy time, cold snare defect protrusion, intraprocedural hematoma formation, and histopathology were recorded prospectively in standardized case report forms and the hospital information system. Medication discontinuation and resumption protocols for patients receiving antithrombotic therapy followed guideline recommendations and individualized cardio-cerebrovascular risk assessments.

Continuous variables are presented as mean ± standard deviation (SD) and categorical variables as *n* (%). Between-group comparisons used Student’s *t* test or the Mann–Whitney *U* test for continuous data and the chi-squared test or Fisher’s exact test for categorical data, as appropriate. Because clip placement was indication-based rather than randomized, between-group comparisons were considered descriptive. No propensity-score matching, weighting, or causal effect modeling was performed because clip placement was protocol-triggered by persistent IPPB.

Exploratory multivariable modeling was restricted to patient-level DPPB due to the small number of events. Firth’s penalized logistic regression was used to reduce bias from rare events. Variables with *P* < 0.20 in univariable analysis were included in the multivariable models. The variable-selection workflow is provided in **Supplementary Table S1** . Adjusted odds ratios (aORs) with 95% profile-likelihood confidence intervals (CIs) are reported. Multicollinearity was assessed using the variance inflation factor (<2 for all retained variables). Statistical analyses were performed using R version 4.4.1 (R Foundation for Statistical Computing, Vienna, Austria).

## Results

### Study Population and Management Algorithm

During the study period, 2403 patients were screened. Of these, 155 did not meet the prespecified polyp eligibility criteria, 70 had lesions suspicious for malignancy, and 2 had inflammatory bowel disease, familial adenomatous polyposis or other syndromic conditions. The final cohort comprised 2176 patients with 4343 eligible 4-9 mm nonpedunculated colorectal polyps ( Fig. 1 ). 30-day clinical outcomes were ascertained from the electronic medical record. Persistent IPPB requiring clips occurred in 315 patients (14.5%) involving 369 lesions (8.5%); the remaining 1861 patients and 3974 lesions had no persistent IPPB and were managed without clipping.

**Fig. 1:**
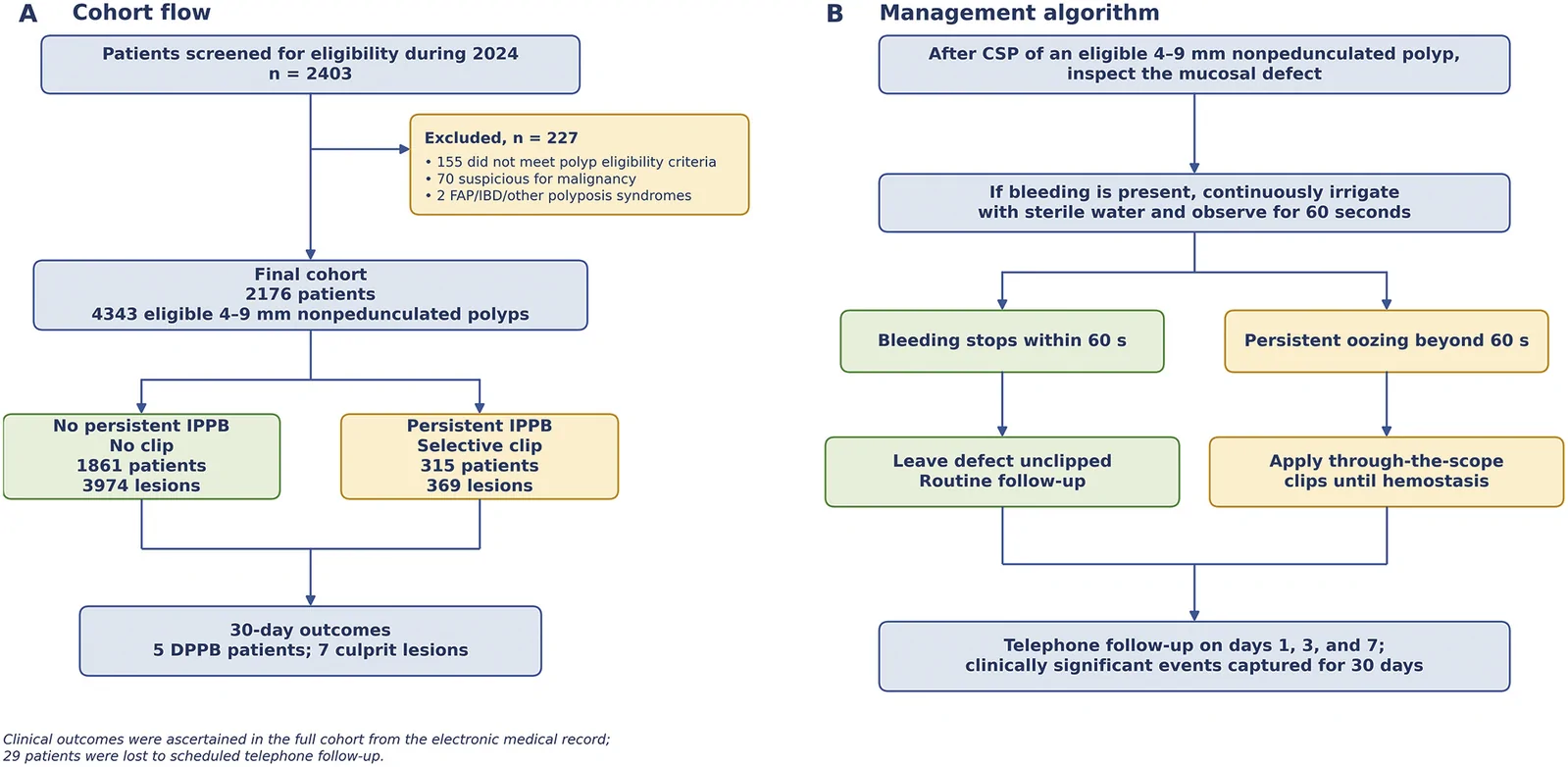
Cohort flow ( **A** ) and standardized hemostasis algorithm ( **B** ). Panel A shows cohort assembly and grouping. Panel B shows the 60-second irrigation-and-observation protocol, with clips reserved for persistent oozing. CSP, cold snare polypectomy; IPPB, immediate post-polypectomy bleeding; DPPB, delayed post-polypectomy bleeding.

### Baseline Characteristics

Patient-level characteristics are summarized in Table 1 . Compared with the no-clip group, the selective-clip group was older (57.20 ± 11.15 vs. 55.05 ± 12.07 years; *P* = 0.002) and had higher prevalences of hypertension, hyperlipidemia, cardiovascular disease, cerebrovascular disease, chronic kidney disease (CKD), and antiplatelet use. Patients in the selective-clip group also had a higher mean number of synchronous polyps (2.72 ± 2.01 vs. 1.94 ± 1.42; *P* < 0.001). No significant differences were observed in sex distribution, body mass index, or bowel preparation quality.

**Table 1:** Patient-level baseline characteristics.

Characteristic	No-clip group ( *n* = 1861)	Selective-clip group ( *n* = 315)	*P* value
Female, *n* (%)	874 (46.96)	137 (43.49)	0.253
Age, mean ± SD, years	55.05 ± 12.07	57.20 ± 11.15	0.002
Body mass index, mean ± SD	24.66 ± 3.44	24.98 ± 3.31	0.115
Hypertension, *n* (%)	395 (21.23)	83 (26.35)	0.042
Diabetes mellitus, *n* (%)	169 (9.08)	38 (12.06)	0.095
Hyperlipidemia, *n* (%)	135 (7.25)	34 (10.79)	0.030
Cardiovascular disease, *n* (%)	74 (4.00)	21 (6.67)	0.031
Cerebrovascular disease, *n* (%)	30 (1.61)	16 (5.08)	<0.001
Chronic liver disease, *n* (%)	11 (0.59)	5 (1.59)	0.056
Chronic kidney disease, *n* (%)	11 (0.59)	6 (1.90)	0.014
Any antiplatelet use, *n* (%)	66 (3.54)	26 (8.26)	<0.001
Any anticoagulant use, *n* (%)	4 (0.22)	2 (0.64)	0.211
BBPS, mean ± SD	7.74 ± 1.01	7.74 ± 0.86	>0.999
Number of polyps per patient, mean ± SD	1.94 ± 1.42	2.72 ± 2.01	<0.001

*BBPS, Boston Bowel Preparation Scale.*

Lesion-level characteristics are shown in Table 2 . Lesion location and Paris morphology differed between groups. Compared with unclipped lesions, clip-treated lesions were larger (6.12 ± 1.32 vs. 5.43 ± 1.17 mm; *P* < 0.001), were more often located in the rectum (10.0% vs. 5.5%), and more frequently had Paris 0-Isp morphology (11.9% vs. 3.5%). Cold snare defect protrusion was less common in clip-treated lesions (30.1% vs. 36.1%; *P* = 0.021), whereas intraprocedural hematoma formation (44.7% vs. 13.1%; *P* < 0.001) and polypectomy time (191.34 ± 130.54 vs. 92.03 ± 46.16 s; *P* < 0.001) were higher. Histopathological category and specimen retrieval failure did not differ significantly between groups.

**Table 2:** Lesion-level characteristics.

Characteristic	Unclipped lesions ( *n* = 3974)	Clip-treated lesions ( *n* = 369)	*P* value
Size, mean ± SD, mm	5.43 ± 1.17	6.12 ± 1.32	<0.001
≤5 mm, *n* (%)	2247 (56.54)	119 (32.25)	
6–9 mm, *n* (%)	1727 (43.46)	250 (67.75)	
Right colon, *n* (%)	1054 (26.52)	103 (27.91)	<0.001*
Transverse colon, *n* (%)	1097 (27.60)	71 (19.24)	
Left colon, *n* (%)	1604 (40.36)	158 (42.82)	
Rectum, *n* (%)	219 (5.51)	37 (10.03)	
Paris 0-Is, *n* (%)	580 (14.59)	58 (15.72)	<0.001†
Paris 0-Isp, *n* (%)	140 (3.52)	44 (11.92)	
Paris 0-IIa/0-IIa+IIc, *n* (%)	3254 (81.88)	267 (72.36)	
Cold snare defect protrusion, *n* (%)	1434 (36.08)	111 (30.08)	0.021
Hematoma formation, *n* (%)	519 (13.06)	165 (44.72)	<0.001
Polypectomy time, mean ± SD, s	92.03 ± 46.16	191.34 ± 130.54	<0.001
Adenoma, *n* (%)	2794 (70.31)	257 (69.65)	0.101‡
Sessile serrated lesion, *n* (%)	65 (1.64)	8 (2.17)	
Traditional serrated adenoma, *n* (%)	16 (0.40)	5 (1.36)	
Nonneoplastic lesion, *n* (%)	1063 (26.75)	94 (25.47)	0.596
Unsuccessful tissue retrieval, *n* (%)	36 (0.91)	5 (1.36)	0.393

* Overall P value for lesion location.

*† Overall P value for Paris morphology.*

*‡ Overall P value for pathological category. Pathological categories are mutually exclusive among retrieved specimens; unsuccessful tissue retrieval is reported separately. Nonneoplastic lesions include hyperplastic polyps, inflammatory polyps, and juvenile polyps.*

### Primary and Secondary Outcomes

At the patient level, DPPB occurred in 5 of 2176 patients (0.23%), including 3 of 1861 patients (0.16%) in the no-clip group and 2 of 315 patients (0.63%) in the selective-clip group ( *P* = 0.154) ( Fig. 2 ; Table 3 ). This numerical difference was not statistically significant and was considered descriptive because clip placement was protocol-triggered rather than randomized. All five DPPB events were confirmed by repeat colonoscopy and successfully managed with endoscopic clipping. Under the prespecified protocol definition, all five events met criteria for severe DPPB because repeat therapeutic endoscopy was performed; however, transfusion was required in only one patient, and no patient required angiographic embolization or surgery. Bleeding occurred 1-4 days after CSP in the no-clip group and 1-2 days after CSP in the selective-clip group. Hematochezia or melena without confirmed DPPB occurred in five patients (0.27%) in the no-clip group and three patients (0.95%) in the selective-clip group. No perforation or post-polypectomy fever occurred.

**Fig. 2:**
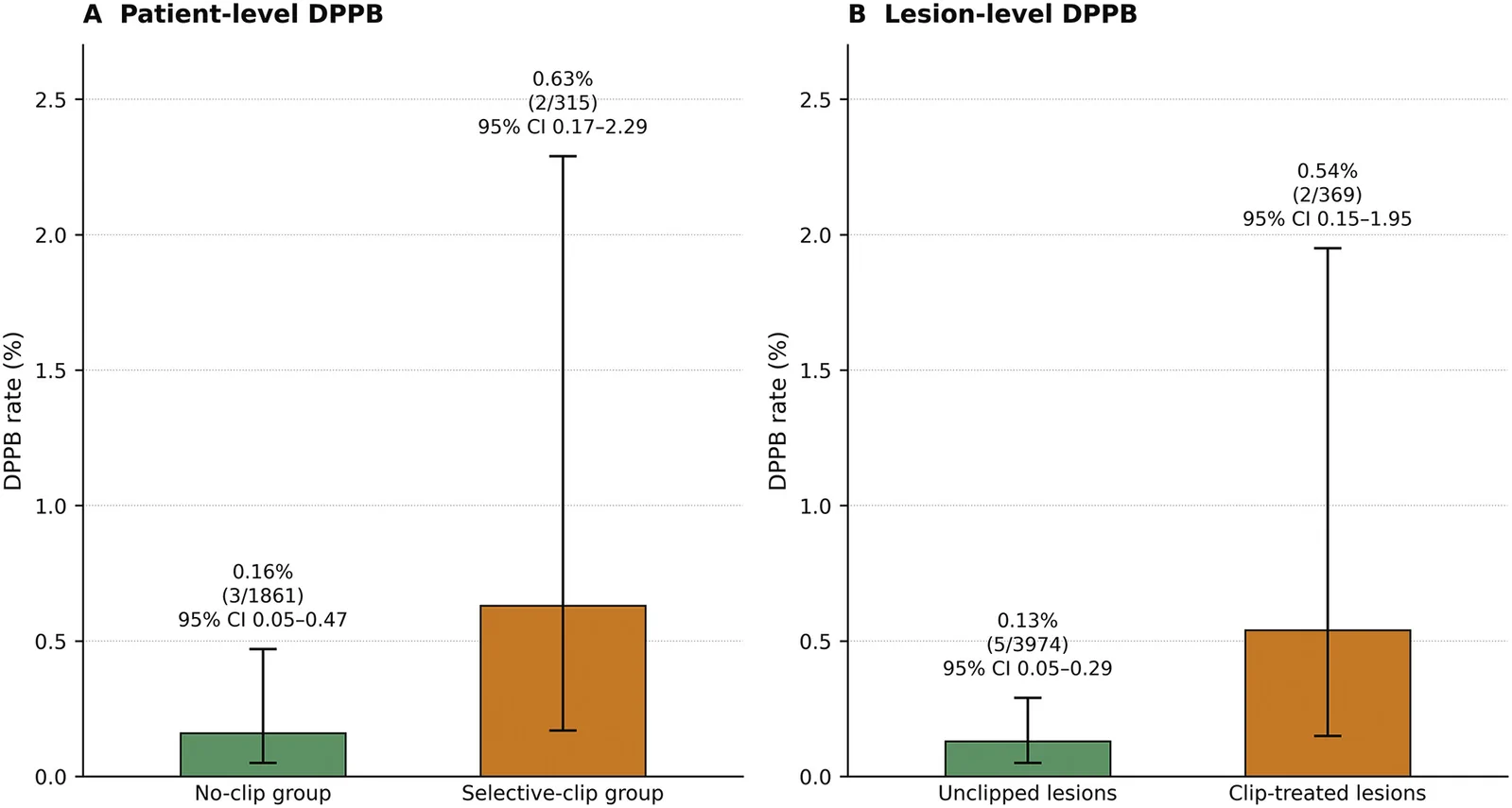
Delayed post-polypectomy bleeding (DPPB) rates under the standardized selective-clip strategy. Patient-level results are shown on the left and lesion-level results on the right. Error bars indicate 95% confidence intervals calculated using the Wilson method.

**Table 3:** Patient-level clinical outcomes.

Outcome	No-clip group ( *n* = 1861)	Selective-clip group ( *n* = 315)	*P* value
Delayed post-polypectomy bleeding, *n* (%)	3 (0.16)	2 (0.63)	0.154
Severe DPPB*, *n* (%)	3 (0.16)	2 (0.63)	0.154
Transfusion required, *n* (%)	1 (0.05)	0	>0.999
Any postprocedural hematochezia or melena without confirmed DPPB, *n* (%)	5 (0.27)	3 (0.95)	0.096
Time to DPPB, range, days	1–4	1–2	NA
Bleeding-related length of stay, range, days	2–6	4–6	NA
Perforation or post-polypectomy fever, *n* (%)	0	0	>0.999

Clinical outcomes were ascertained in the full cohort from the electronic medical record; DPPB, delayed post-polypectomy bleeding. * Severe DPPB was prespecified as DPPB associated with hemoglobin drop >20 g/L, transfusion, repeat therapeutic endoscopy, angiographic intervention, or surgery; all five confirmed DPPB events met this protocol definition because repeat endoscopic hemostasis was performed.

At the lesion level, DPPB occurred in 7 of 4343 lesions (0.16%), including 5 of 3974 unclipped lesions (0.13%) and 2 of 369 clip-treated lesions (0.54%) ( *P* = 0.219) ( Fig. 2 ). Among the five patients with DPPB, three had one culprit lesion and two had two culprit lesions, resulting in seven adjudicated lesion-level events in the secondary analysis. Culprit stigmata consisted of adherent clot alone in four lesions, adherent clot with an exposed vessel in one lesion, and adherent clot with active oozing in two lesions ( Table 4 ).

**Table 4:** Adjudicated culprit lesions in the five patients with delayed post-polypectomy bleeding.

Patient	Group	Day of DPPB	Culprit lesions, *n*	Culprit lesion summary	Repeat-colonoscopy stigmata and management
1	No-clip	2	2	9 mm hepatic flexure IIa hyperplastic polyp; 6 mm descending colon IIa adenoma	Adherent clot + exposed vessel at one site; adherent clot at one site; clips; no transfusion
2	Selective-clip	2	1	8 mm transverse colon IIa adenoma	Adherent clot at previously clip-treated defect; clips; no transfusion
3	Selective-clip	1	1	6 mm sigmoid colon Isp adenoma	Adherent clot at previously clip-treated defect; clips; no transfusion
4	No-clip	4	1	6 mm ascending colon IIa adenoma	Adherent clot; clips; no transfusion
5	No-clip	1	2	6 mm transverse colon Is adenoma; 4 mm transverse colon IIa lesion not retrieved	Adherent clot + active oozing at both sites; clips; transfusion

*Culprit lesions were adjudicated at repeat endoscopy on the basis of predefined stigmata of recent hemorrhage: active bleeding, exposed vessel, or an adherent clot persisting despite targeted irrigation. All five DPPB patients underwent repeat colonoscopy. Two endoscopists reviewed each case independently, with a third reviewer available to resolve disagreements. DPPB, delayed post-polypectomy bleeding.*

### Exploratory Risk-factor Analysis

Because only five patient-level DPPB events occurred, all risk-factor analyses were considered exploratory and hypothesis-generating. In exploratory patient-level Firth models, CKD was retained in the final model for the no-clip cohort (aOR 23.35, 95% CI 1.20–369.70; *P* = 0.040), whereas resection of ≥5 synchronous polyps was retained in the selective-clip cohort (aOR 17.60, 95% CI 1.20–1096.90; *P* = 0.037) ( Table 5 ). **Supplementary Table S1** details the univariable screening and the variables retained in or excluded from each multivariable model.

**Table 5:** Exploratory patient-level Firth regression signals for delayed post-polypectomy bleeding.

Cohort	DPPB events	Retained variable	Adjusted odds ratio (95% CI)	*P* value
No-clip group ( *n* = 1861)	3	Chronic kidney disease	23.35 (1.20–369.70)	0.040
Selective-clip group ( *n* = 315)	2	≥5 synchronous polyps	17.60 (1.20–1096.90)	0.037

*The very wide confidence intervals reflect the small number of DPPB events; these findings are exploratory and hypothesis-generating only. Variable selection is detailed in* ***Supplementary Table S1.***

### Antithrombotic Continuation Subgroup

Twenty-one patients continued antithrombotic therapy through the procedure (14 in the no-clip group and 7 in the selective-clip group). One patient continuing warfarin developed DPPB after clip-treated persistent IPPB; no DPPB occurred in the remaining 20 patients.

## Discussion

Persistent IPPB after CSP represents a practical intraprocedural management challenge because a standardized approach is lacking. Reported IPPB rates vary substantially across studies, largely owing to heterogeneous definitions and predominant lesion-level reporting. To address this gap, our center adopted a standardized 60-second irrigation-and-observation rule, with clips reserved for persistent oozing, and this prospective cohort was designed to describe DPPB outcomes under that protocol. Three main observations emerged. First, overall patient-level DPPB after CSP of 4-9 mm polyps was rare (0.23%). Second, only a minority of lesions (8.5%) required clip application after the standardized 60-second assessment. Third, all DPPB events were endoscopically confirmed and successfully managed with endoscopic therapy.

Our DPPB rates are consistent with contemporary CSP series. In a Taiwanese screening cohort, DPPB occurred in approximately 0.1% of patients after implementation of CSP. 3 In a Japanese multicenter study, DPPB occurred in 26 of 18,007 lesions (0.14%). 4 A recent multicenter Chinese retrospective study of 10,650 patients reported a DPPB rate of 0.24% and identified hypertension, hyperlipidemia, antithrombotic therapy, hematoma, cold snare defect protrusion, wound bleeding grade, and morphology as candidate predictors. 5 Taken together with our data, the central message is that delayed bleeding after small-polyp CSP is uncommon even when immediate oozing is encountered during the procedure.

Our lesion-level persistent IPPB rate of 8.5% is consistent with the expected bleeding pattern after CSP because immediate oozing after cold transection is common, whereas only a subset persists long enough to warrant endoscopic hemostasis. ESGE considers bleeding lasting longer than 60s and/or requiring endoscopic intervention to be clinically significant. 2 Within that framework, the 60-second irrigation-and-observation rule provided a reproducible threshold for clip application. Importantly, this protocol was applied only to defects with visible bleeding, so its practical trade-off is additional observation time for bleeding defects rather than universal prolongation of all CSP procedures.

The numerically higher, but not statistically significant, DPPB rate observed in the selective-clip group should be interpreted in the context of the standardized algorithm and the non-exchangeability of the two protocol-defined branches. Persistent IPPB identified a subgroup with greater baseline bleeding vulnerability: patients in this group were older, had more comorbidities and antiplatelet use, and had larger or more morphologically complex lesions. Lesion-level findings in this branch also indicated greater procedural bleeding severity, including more frequent intraprocedural hematoma formation and longer polypectomy times. Therefore, the between-group difference is best interpreted as reflecting differences in baseline bleeding risk rather than the efficacy of clipping. Because more than one resection site could meet culprit criteria in the same patient, lesion-level findings should also be interpreted as secondary to the patient-level analysis.

The exploratory risk-factor signals in our cohort should be interpreted with similar caution. CKD emerged as a signal in the no-clip cohort, which is biologically plausible given platelet dysfunction and uremia-related hemostatic abnormalities, and is directionally consistent with recent large-scale evidence linking CKD to DPPB after colorectal polypectomy. 14 In the selective-clip cohort, ≥5 synchronous polyps emerged as a signal, suggesting that total mucosal injury burden may matter once immediate bleeding has already occurred. However, these models were based on only five patient-level events, with very wide CIs, and should be viewed strictly as hypothesis-generating signals rather than as established predictors. These estimates are therefore insufficient for reliable clinical risk stratification and require external validation.

The antithrombotic subgroup likewise requires restraint. Only 21 patients continued antithrombotic therapy, and only one bleeding event occurred (in a patient continuing warfarin). Although event counts were very small, our observations are broadly consistent with prior CSP studies showing low but nonzero DPPB risk in antithrombotic users and uncertain incremental benefit of prophylactic clipping in this population. 15 16 17 18 Future comparative studies focused specifically on persistent IPPB in antithrombotic users would be valuable.

This study has several notable features. First, it evaluated a standardized 60-second irrigation-and-observation algorithm for persistent IPPB rather than relying solely on operator preference, providing a practical and reproducible management framework for everyday CSP. Second, it combined patient- and lesion-level analyses with predefined culprit-lesion adjudication, allowing more granular interpretation of bleeding source, event pattern, and risk signals.

This study also has several limitations. First, it was conducted at a single tertiary center in China, which may limit generalizability to other practice settings. Second, clip application was not randomized; accordingly, the observed difference between groups cannot be interpreted as a causal estimate of clip efficacy. Third, only five patient-level DPPB events occurred, limiting the precision of multivariable analyses and subgroup comparisons. Fourth, lesion-level attribution was secondary and may be imperfect when multiple post-CSP defects showed recent hemorrhagic stigmata in the same patient. Fifth, exact irrigation volume, detailed position-change maneuvers, and formal semiquantitative bleeding-severity grading were not prospectively standardized or recorded. Finally, although forced CSP has been associated with cold snare defect protrusion and incomplete polyp resection, 19 this maneuver was not separately recorded in our cohort; therefore, its potential influence on defect protrusion, hematoma formation, completeness of resection, and bleeding outcomes cannot be excluded.

In conclusion, DPPB after CSP of 4-9 mm nonpedunculated colorectal polyps was rare under a standardized selective-clip protocol that reserved clips for persistent IPPB after 60s of irrigation. All confirmed bleeding events were successfully managed endoscopically. These findings suggest that a standardized approach to persistent IPPB is feasible as a management framework, but they do not establish a protective effect of clipping. Further comparative studies are needed before routine implementation can be recommended.

## Data Availability

The data underlying this article will be shared on reasonable request to the corresponding author.

## Supplementary Material

Ergänzendes Material Supplementary Material
