# Supplementary material for: Standardized Selective Clipping for Persistent Immediate Bleeding after Cold Snare Polypectomy of Small Colorectal Polyps: A Prospective Cohort Study
Source: Endosc Int Open. 2026 Sep 30;14:a29377891. doi: 10.1055/a-2937-7891 (PMC13643731; doi:10.1055/a-2937-7891)
Supplement: Supplementary file 1 [file 10-1055-a-2937-7891_29499515.pdf]

Supplementary Table S1. Variable-selection workflow for exploratory patient-level Firth regression models.

Variable with univariable (P < 0.20)	Univariable P value	Entered into multivariable Firth model	Final model status
No-clip group (n = 1861; DPPB events = 3)			
Age >65 years	0.081	Yes	Excluded
Cardiovascular or cerebrovascular disease	0.002	Yes	Excluded
Chronic liver disease	0.148	Yes	Excluded
Chronic kidney disease	<0.001	Yes	Retained
Any antiplatelet use	0.027	Yes	Excluded
Any anticoagulant use	0.085	Yes	Excluded
Selective-clip group (n = 315; DPPB events = 2)			
Age >65 years	0.008	Yes	Excluded
BMI >25 kg/m²	0.070	Yes	Excluded
Cardiovascular or cerebrovascular disease	0.164	Yes	Excluded
Composite metabolic comorbidity (hypertension, diabetes mellitus, and/or hyperlipidemia)	0.027	Yes	Excluded
Chronic kidney disease	0.010	Yes	Excluded
Any anticoagulant use	0.002	Yes	Excluded
≥5 synchronous polyps	0.030	Yes	Retained

DPPB, delayed post-polypectomy bleeding; BMI, body mass index. Only variables with univariable P < 0.20 in branch-specific patient-level analyses are shown. All listed variables were entered into exploratory multivariable Firth models. Final adjusted estimates are shown in Table 5.
